# A Retrospective Analysis of the Impact of Myomectomy on Survival in Uterine Sarcoma

**DOI:** 10.1371/journal.pone.0148050

**Published:** 2016-02-01

**Authors:** Zhenzhen Gao, Li’an Li, Yuanguang Meng

**Affiliations:** 1 Department of Obstetrics and Gynecology, General Hospital of Armed Police Forces, Beijing, China; 2 Department of Obstetrics and Gynecology, Chinese PLA General Hospital, Beijing, China; Duke University Medical Center, UNITED STATES

## Abstract

Laparoscopic myomectomy is a minimally invasive, conservative surgical approach commonly used for the treatment of uterine fibroids. However, there is a lack of effective means to distinguish the nature of uterine tumors prior to surgery. The impact of fibroid morcellation during laparoscopic surgery on the dissemination of cancerous uterine fibroids and long-term survival of patients has gained increasing attention. A retrospective cohort study was conducted to analyze the impact of different surgical approaches on recurrence-free survival (RFS) and overall survival (OS) in patients with a postoperative pathological diagnosis of uterine sarcoma at a single medical center. Patients who underwent the first surgery for uterine fibroids (confined to the uterus) and had a postoperative pathological diagnosis of uterine sarcoma were selected in the Chinese PLA General Hospital from January 2005 to January 2014. Based on the use of fibroid morcellation, the subjects were divided into fibroid morcellation (FM) and total hysterectomy (TH, non-morcellation) groups. Follow-up outcomes, including RFS and OS times, were observed. In total, 59 patients were included, with 30 cases in the FM group and 29 cases in the TH group. There were no significant differences in RFS and OS time between the two groups (RFS: P = 0.16, OS: P = 0.09). Multivariate correlation analysis showed that the impact of a higher grade level on RFS and OS was nearly 2-fold the impact of a lower grade level (RFS: P = 0.04, odds ratio (OR) = 1.97; OS: P = 0.03, OR = 2.29). Intraoperative morcellation, postoperative adjuvant therapy, age, tumor size, FIGO stage, and surgical approach were not risk factors affecting RFS and OS. Fibroid morcellation during laparoscopic surgery (including laparoscopic, transvaginal and transabdominal approaches) had no significant impact on RFS and OS time in patients. However, the 5-year RFS and OS rates were both lower in the FM group than in the TH group. Grade level was a significant risk factor for the prognosis of patients with uterine sarcoma.

## Introduction

Uterine sarcomas are a rare type of gynecologic cancer, accounting for ~3% of uterine malignancies. Owing to its high grade of malignancy and a lack of specific clinical manifestations, early diagnosis of uterine sarcoma remains difficult, with a poor prognosis. Common pathological types of uterine sarcoma include leiomyosarcoma (LMS), endometrial stromal sarcoma (ESS), and mixed malignant mesodermal tumor (MMMT). Among the three pathological types, LMS is the most common uterine sarcoma [1, 2] and may have an origin in malignant uterine leiomyomas (also known as fibroids). Following total hysterectomy (TH) for uterine fibroids, the prevalence of uterine LMS and ESS in postoperative pathological specimens are approximately 0.23–0.49% [3, 4] and 0.23% [5], respectively. Owing to the rapid development of laparoscopic techniques, laparoscopic myomectomy has become a minimally invasive, conservative surgical approach commonly used for the treatment of uterine fibroids. However, there have been international reports [6–8] indicating that myomectomy has a significant impact on long-term survival in uterine LMS. Retrospective analyses by Park [7] and George et al. [8] showed that myomectomy affected recurrence-free survival (RFS) and overall survival (OS) in patients with uterine LMS. Moreover, a study by Bogani et al. [9] showed that intraoperative myomectomy increased the recurrence and mortality rates of uterine LMS. The US Food and Drug Administration (FDA) released a warning in 2014 [10] that the application of a laparoscopic electric morcellator in patients with unpredictable uterine sarcoma can increase the risk of dissemination of tumor tissue in the abdominopelvic cavity, particularly affecting patient long-term survival rates. Moreover, owing to a lack of effective means for preoperative differentiation of the malignancy of uterine fibroids [11, 12], the use of the electric morcellator is not recommend during laparoscopic myomectomy. In China, despite a late start, the surgical approach of laparoscopic myomectomy has been applied extensively, primarily owing to its distinct advantages relative to traditional transabdominal myomectomy with respect to abdominal incision, intraoperative bleeding, bed rotation rate, and other aspects. Moreover, laparoscopic myomectomy is associated with a lower tumorigenesis rate in uterine fibroids. Therefore, how exactly to choose when to perform laparoscopic myomectomy has become a problem in clinical treatment. Clinical trials about the impact of laparoscopic myomectomy on long-term survival of patients are currently rare in China. The present study provided a retrospective analysis on the incidence and long-term follow-up of uterine sarcoma following laparoscopic myomectomy for uterine fibroids. Furthermore, the impact of laparoscopic myomectomy on the long-term survival time of patients was analyzed to guide clinical applications.

## Subjects and Methods

### Ethics Statement

Before the study began, the study was approved by the Ethics Committee of Chinese PLA General Hospital. All patients provided written informed consent. Transcripts of the important information obtained by the interviews were written down.

### Subjects

In total, 59 patients were included in this study. The included patients all had a preliminary diagnosis of uterine fibroids (confined to the uterus), their first surgery occurred after admission (including laparoscopic, transvaginal and transabdominal approaches), and a postoperative pathological diagnosis of uterine sarcoma was made in the Chinese PLA General Hospital from January 1, 2005 to January 1, 2014. We excluded patients with other concomitant malignant tumors and patients in whom specimen retrieval bags were used during laparoscopic surgery. Based on the use of fibroid morcellation, the subjects were divided into exposure-fibroid morcellation (FM) and non-exposure-total hysterectomy (TH, non-morcellation) groups. Fibroids were morcellated laparoscopically using a rotary cutting device or transvaginally and transabdominally using a scalpel. The statistics of the basic clinical data are shown in Table 1.

**Table 1:** Statistics of basic clinical data.

	Group (cases)			
	Fibroid morcellation	Total hysterectomy	*χ*^2^ (*t*)	*P*
Age (years)	45.07±10.85	50.72±14.34	2.83	0.09
Menopause			6.12	0.01
Yes	5(16.7%)	15(51.7%)		
No	25(83.3%)	14(48.3%)		
Tumor size (cm)	7.50±2.74	6.11±2.61	2.01	0.05
Pathological type				0.09
Uterine leiomyosarcoma	11(36.7%)	6(20.7%)		
Endometrial stromal sarcoma	16(53.3%)	16(55.2%)		
Mixed malignant mesodermal tumor	3(10.0%)	7(24.1%)		
Surgical approach				0.32
Laparoscopic	6(20.0%)	5(17.2%)		
Transabdominal	16(53.3%)	12(41.4%)		
Transvaginal	8(26.7%)	12(41.4%)		
FIGO stage				0.16
I	19(63.3%)	23(79.3%)		
II	6(20.0%)	4(13.8%)		
III	5(16.7%)	2(6.9%)		
Grade level				0.70
1	19(63.3%)	16(55.2%)		
2	3(10.0%)	6(20.7%)		
3	8(26.7%)	7(24.1%)		
Bilateral salpingo-oophorectomy			0.42	0.52
Yes	14(46.7%)	18(62.1%)		
No	16(53.3%)	11(37.9%)		
Adjuvant therapy				0.39
No	13(43.3%)	12(41.4%)		
Chemotherapy (incl. hormone therapy)	9(30.0%)	4(13.8%)		
Radiotherapy	6(20.0%)	9(31.0%)		
Chemotherapy + radiotherapy	2(6.7%)	4(13.8%)		
Recurrence			0.83	0.36
Yes	15(50%)	11(37.9%)		
No	15(50%)	18(62.1%)		
Recurrence(FIGO stage I)			2.21	0.33
Intra-abdominal	6(66.7%)	5(71.4%)		
Extra-abdominal	1(11.1%)	2 (28.6%)		
Peritoneal dissemination	2(22.2%)	0(0%)		

Age: The patients in the FM group were 30 to 55 years of age, with an average age of 45 years; the patients in the TH group were 26 to 74 years of age, with an average age of 50 years; there was no significant difference in age between the two groups (P = 0.09). Menopause: Menopausal patients accounted for 16.7% in the FM group and 51.7% in the TH group, with a significant difference between the groups (P = 0.01). Tumor size: The mean tumor diameters of the patients were ~7.50 cm in the FM group and ~6.11 cm in the TH group, showing no significant difference between the groups (P = 0.05).

### Methods

Evaluation criteria for follow-up observations: RFS—time from the start date to a clear diagnosis of uterine sarcoma recurrence by the first imaging examination; if no recurrence was detected, the observation period lasted to the end date. OS—time from the start date to death; if no death occurred, the observation period lasted to the end date. Start date: the day of the first surgery after admission to the hospital; end date: January 31, 2015. Exclusion criteria: Death refers to death directly caused by uterine sarcoma; otherwise, death caused by other causes was excluded.

Continuous data were analyzed using an independent-samples t-test. Categorical and ordinal data were analyzed using an independent-samples nonparametric test. The two groups were compared in terms of RFS and OS time using Kaplan-Meier survival curves. Multivariate correlation analysis was performed using Cox regression. Statistical analysis was performed in SPSS 19.0. Differences were considered statistically significant at P < 0.05.

## Results

### Statistics of general clinical data

In total, there were 3,986 patients with a primary diagnosis of uterine fibroids (confined to the uterus) who underwent their first surgical treatment after admission to our hospital from January 01, 2005 to 01 May 2014. Among them, 59 cases had a postoperative pathological diagnosis of uterine sarcoma, accounting for an prevalence of 1.48%. There were 17 cases with LMS (28.8%), 32 cases with ESS (54.2%), and 10 cases with MMMT (16.9%). According to the 2009 FIGO staging system for uterine sarcomas, there were 42 stage I cases (71.2%), 10 stage II cases (16.9%), and 7 stage III cases (11.9%).

The prevalence of uterine sarcoma was analyzed in terms of “different surgical approaches.” In total, 843 patients underwent laparoscopic myomectomy, among whom 6 cases (0.71%) had a postoperative pathological diagnosis of uterine sarcoma; 1,315 patients underwent abdominal or transvaginal myomectomy, among whom 24 cases (1.83%) had a postoperative pathological diagnosis of uterine sarcoma; 1,828 patients underwent total hysterectomy, among whom 29 cases (1.56%) had a postoperative pathological diagnosis of uterine sarcoma.

Statistics on the basic clinical data also included age, menopause, tumor size measured in the last preoperative imaging examination (e.g., ultrasound and MRI), intraoperative bilateral salpingo-oophorectomy, postoperative adjuvant therapy, grade level, and postoperative recurrence (Table 1).

With respect to surgical approach, the transabdominal approach accounted for a high proportion in the FM and TH groups (53.3% *vs*. 41.4%), while the laparoscopic approach accounted for a small proportion (20.0% *vs*. 17.2%). Moreover, a high proportion of patients received no postoperative adjuvant therapy in the two groups (43.3% *vs*. 41.4%). There were 9 cases of recurrence in the FM group and 7 in the TH group of patients with FIGO stage I(47.4% *vs*. 30.4%). The statistical data showed that there were no significant differences between the two groups in terms of frequency distribution of histological type, FIGO stage, grade level, bilateral salpingo-oophorectomy, postoperative adjuvant therapy, surgical approach, or recurrence (P > 0.05).

### Comparison of RFS and OS times between groups (Figs 1 and 2)

**Fig 1:**
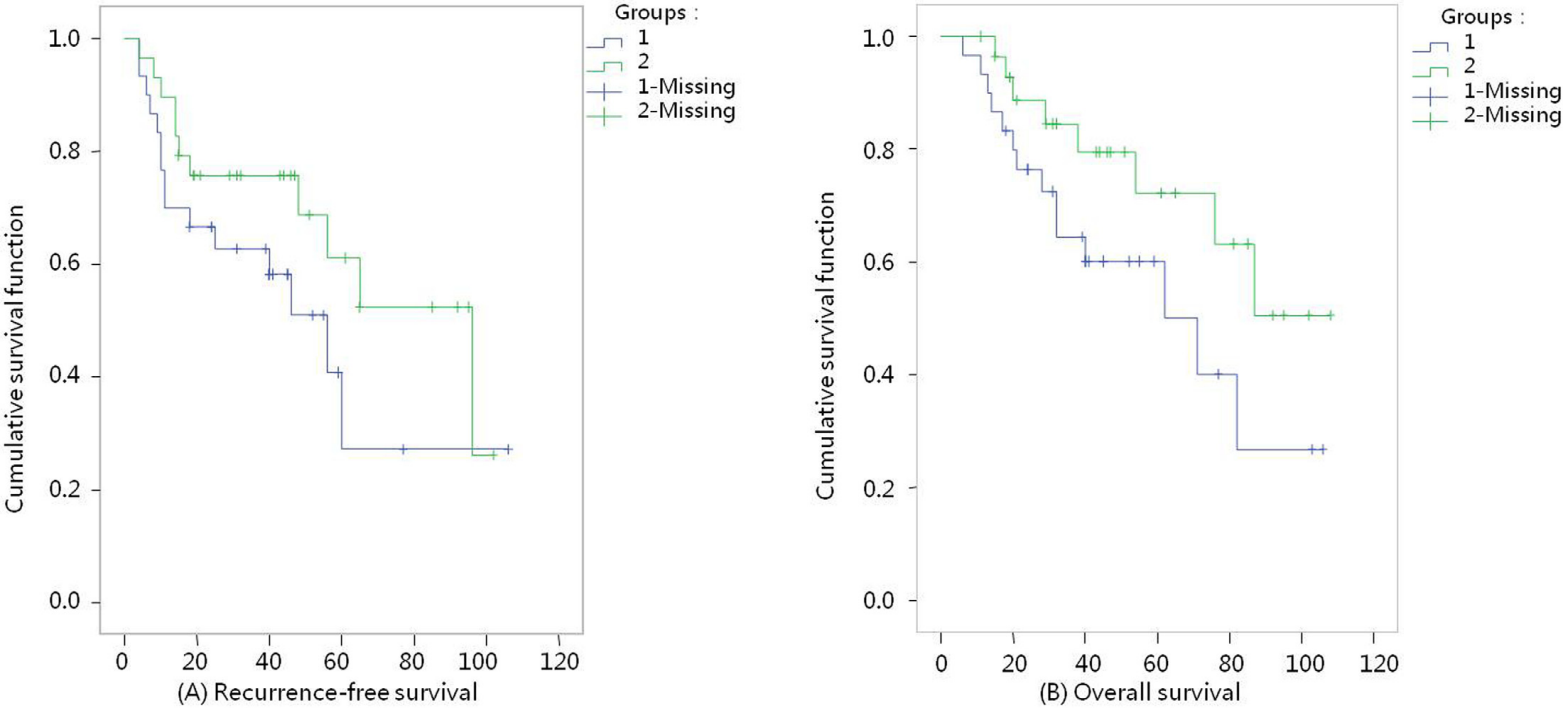
Survival curves (l-Fibroid morcellation group, 2-Total hysterectomy group). (A) Recurrence-free survival K-M curves, P = 0.16. (B) Overall survival K-M curves, P = 0.09.

**Fig 2:**
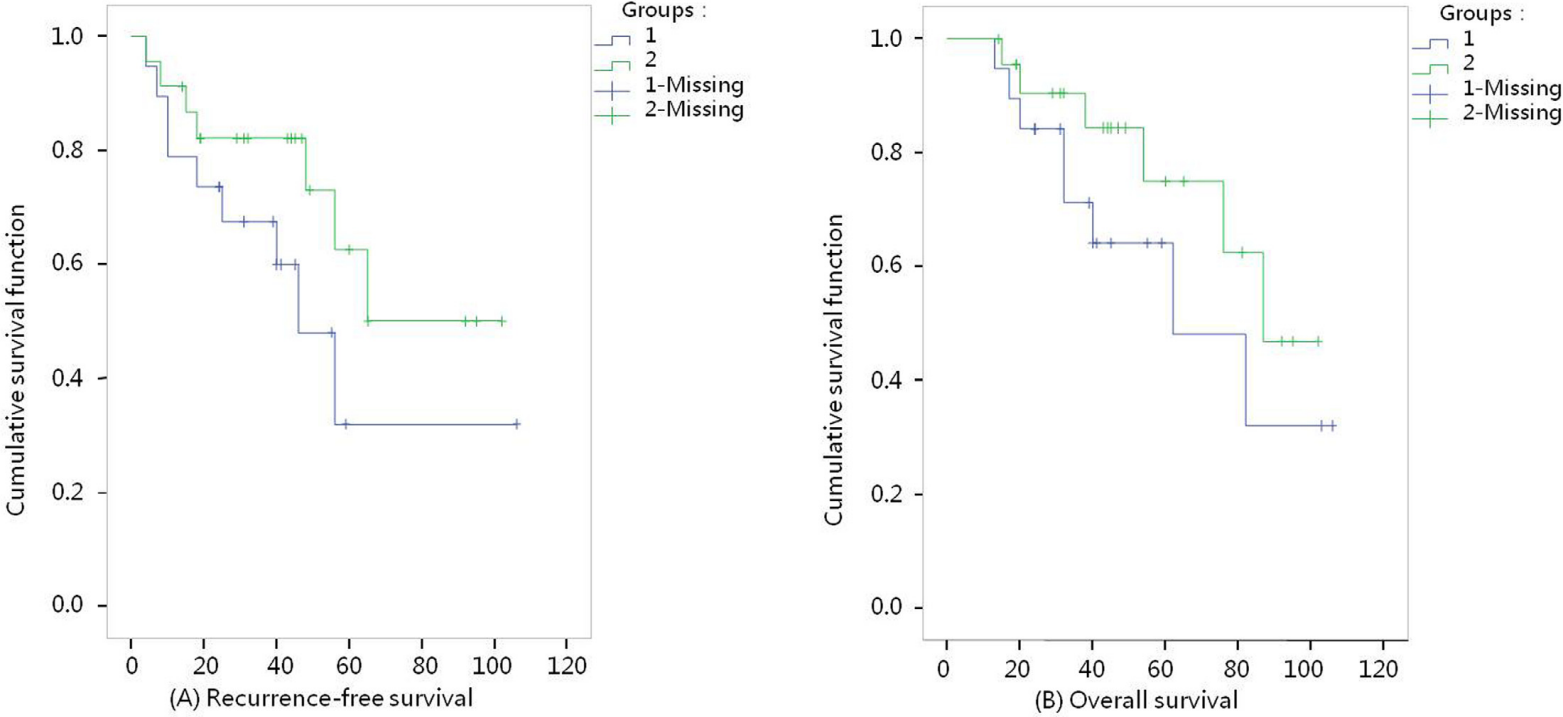
Survival curves with FIGO stage I (l-Fibroid morcellation group, 2-Total hysterectomy group). (A) Recurrence-free survival K-M curves, P = 0.15. (B) Overall survival K-M curves, P = 0.25.

Fig 1(A) RFS curves: In the FM group, the shortest follow-up time was 4 months and the longest was 106 months, with an average RFS time of 52 months; in the TH group, the follow-up time lasted 4 to 102 months, with an average RFS time of 56 months. The median RFS time lasted 66 months in the FM group and 90 months in the TH group. A comparative analysis revealed that the *χ*^2^ value was 2.01 (P = 0.16), indicating no statistically significant difference in RFS between the two groups. For the patients with FIGO stage I, the median RFS time lasted 46 months in the FM group. A comparative analysis revealed that the *χ*^2^ value was 2.11 (P = 0.15), indicating no statistically significant difference in RFS between the two groups (Fig 2(A) RFS curves).

Fig 1(B) OS curves: In the FM group, the shortest follow-up time was 6 months and the longest was 106 months, with an average OS time of 58 months; in the TH group, the follow-up time lasted 15 to 108 months, with an average OS time of 60 months. The median OS time in the FM group lasted 69 months. Comparison between the two groups showed that the χ^2^ value was 2.86 (P = 0.09), indicating no significant difference in OS between the two groups. For the patients with FIGO stage I, the median OS time lasted 62 months in the FM group and 87 months in the TH group. A comparative analysis revealed that the χ^2^ value was 1.31 (P = 0.25), indicating no statistically significant difference in OS between the two groups (Fig 2(B) OS curves).

### Comparison of RFS and OS rates between groups (Table 2)

**Table 2:** Impact of fibroid morcellation and laparoscopic surgery on survival rates.

Group	Subgroup	Recurrence-free survival rate					Overall survival rate				
		1 year	3 year	5 year	χ²	P	1 year	3 year	5 year	χ²	P
Morcellation	Fibroid morcellation	96.7%	77.2%	24.1%	2.01	0.16	96.7%	79.9%	37.8%	2.86	0.09
	Total hysterectomy	96.5%	70.5%	43.6%			96.6%	73.8%	43.1%		
Laparoscopy	Fibroid morcellation	66.7%	41.7%			0.18	83.3%	66.7%			0.53
	Total hysterectomy	80.0%	60.0%	20.0%			100.0%	80.0%			

The 5-year RFS and OS rates were both lower in the FM group versus the TH group (RFS: 24.1% *vs*. 43.6% and OS: 37.8% *vs*. 43.1%), whereas the 1- and 3-year survival rates were similar in the two groups. A separate analysis of the impact of the laparoscopic approach on survival rates revealed that the 1- and 3-year RFS rates for patients were 66.7% and 41.7%, respectively, in the laparoscopy-FM group; the corresponding OS rates were 83.3% and 66.7%, respectively. In the laparoscopy-TH group, the 1-, 3-, and 5-year RFS rates were 80.0%, 60.0%, and 20.0%, respectively; the 1- and 3-year OS rates were 80.0% and 100%. There was no significant difference in the RFS or OS rate between the two groups (P = 0.18 and P = 0.53, respectively).

### Multivariate correlation analysis of prognostic factors (Table 3)

**Table 3:** Multivariate survival analysis of recurrence-free and overall survival.

	Recurrence-free survival			Overall survival		
	P	Odds ratio	95% confidence interval	P	Odds ratio	95% confidence interval
Morcellation	0.29	1.66	0.65–4.23	0.19	2.21	0.67–7.27
Age	0.13	1.04	0.99–1.11	0.07	1.07	1.00–1.15
Tumor size	0.64	1.04	0.88–1.22	0.28	1.11	0.92–1.33
FIGO stage	0.99	1.00	0.51–1.96	0.74	0.87	0.37–2.02
Grade level	0.04	1.97	1.03–3.79	0.03	2.29	1.10–4.77
Adjuvant therapy	0.88	1.03	0.67–1.61	0.88	0.96	0.56–1.64
Surgical approach	0.64	0.85	0.43–1.67	0.18	0.58	0.26–1.28

Fibroid morcellation, age, tumor size, FIGO stage, adjuvant therapy, and surgical approach were not risk factors affecting RFS and OS in patients with uterine sarcoma (P > 0.05 for all). Grade level was a risk factor affecting RFS and OS in patients with uterine sarcoma (P = 0.04 and P = 0.03, respectively). The risk of a high grade level (i.e., poor differentiation) shortening the ORS and OR was approximately 2-fold the risk of a low grade level shortening the ORS and OR (odds ratio (OR) = 1.97, 95% confidence interval (CI) 1.03–3.79; OR = 2.29, 95% CI 1.10–4.77). Grade level was a significant risk factor affecting the prognosis of patients with uterine sarcoma. There is no relation between the grade and stage state of the tumors.

## Discussion

Laparoscopic myomectomy has become a minimally invasive, conservative surgical approach commonly used in the treatment of uterine fibroids. In this paper, we chose the patients with a primary diagnosis of uterine fibroids (confined to the uterus) who underwent their first surgical treatment. In the FM group, pathological results with 11 patients were uterine sarcoma after their first surgical treatment, then, these patients underwent reoperation. According to the post-reoperative pathological findings (positive lymph nodes or metastatic lesions in the fallopian tube), these patients had FIGO stage II or III tumors. However, all of these 11 patients had no abnormal discovery before surgery. Compared with traditional transabdominal myomectomy, laparoscopic myomectomy exhibits obvious advantages in reducing intraoperative bleeding, shortening operative times and hospital stays, increasing bed turnover rate, and mitigating abdominal incision pain [13, 14]. The prevalence rate of uterine sarcoma in the laparoscopic myomectomy group was lower than the other two groups, and the analysis of the reasons may be related to the limitations of laparoscopic surgery. It was suggested that the large and rapid growth uterine fibroids may be more likely to be removed by trans-vaginal or trans-abdominal approaches. Besides, in this paper, the other two groups had little difference in the prevalence rate of uterine sarcoma. This laparoscopic approach adopts a high-speed rotatory electric morcellator to cut the fibroid and then remove the fragments from the abdominopelvic cavity through a small abdominal incision. This so-called “uterine morcellation or tumor morcellation” may cause dissemination and seeding of the tumor in the abdominopelvic cavity and abdominal incision. Park [7] found that tumor recurrence, as peritoneal sarcomatosis, was significantly more frequent in patients with apparently early uterine LMS who did than did not undergo tumor morcellation. In our follow-up cases, the recurrence of the patients was re-operated, and can be seen in the case of a typical tumor spread. The possible reasons for the dissemination and seeding of the tumor include the following [15, 16]: small tumor fragments are produced from the rotary cutting of fibroids, and small nebulized tumor tissue and cells are generated by the high-speed rotating blade; these materials are shed onto the surface of the abdominopelvic cavity and continue to grow; tumor tissue and cells are compressed and scattered due to rotary cutting and pneumoperitoneum pressure, leading to decentralized growth and tumor recurrence; the difficulty of pathological sampling is increased after the morcellation of tumor tissue, and in cases of malignant tumors, it may lead to a missed diagnosis or to a misdiagnosis and delay in treatment time. Some scholars [17] believe that if uterine sarcoma is detected by pathological diagnosis following laparoscopic myomectomy, it will affect the long-term survival in patients. However, the present retrospective cohort study revealed that fibroid morcellation did not affect RFS or OS in 59 patients with uterine sarcoma in our hospital (P > 0.05). Likewise, the use of morcellation during laparoscopic surgery resulted in no significant difference in patient survival. Multivariate analysis showed that neither fibroid morcellation nor surgical approach was a risk factor affecting RFS and OS. The postoperative pathological grade level was a significant risk factor affecting RFS and OS. However, it is worth noting that the 5-year RFS and OS rates were both lower in the FM group *vs*. the TH group.

This study suggests that laparoscopic fibroid morcellation has a limited impact on the long-term survival of patients who have a postoperative diagnosis of uterine sarcoma. Given the low prevalence of uterine sarcoma and the low probability of detecting malignancy following surgical treatment for benign fibroids (~1.48% in this study), laparoscopic myomectomy remains the conservative treatment option for the majority of benign uterine fibroids. In particular, for young patients, laparoscopic myomectomy prevents the formation of pelvic adhesions and reduces the prevalence of infertility [18] compared with the transabdominal and transvaginal approaches. Because transabdominal fibroid morcellation is associated with the risk of tumor seeding at the abdominal incision [19], it is necessary to fully evaluate and choose the appropriate approach prior to surgery in patients with rapid growth of uterine fibroids and rich blood flow signals on ultrasound or MRI (indicating higher probability of malignancy) to avoid the negative impact potentially brought about by fibroid morcellation (including during laparoscopic, transvaginal and transabdominal approaches). For patients undergoing myomectomy, carefully removing residual tumor fragments and repeated peritoneal washings during surgery can reduce the chance of residual tumor. Theoretically, the patients who undergone morcellation with specimen retrieval bags, might have had a different survival (include DFS and OS) [20], but in our cases, there are few patients with specimen retrieval bags, therefore, we were unable to do effective statistical comparison. Recently, the FDA has recommended that the use of the morcellator with specimen retrieval bags during myomectomy [21] may help to prevent tumor seeding and dissemination. However, the long-term efficacy of this recommended protocol has not been verified clinically.

## Supporting Information

S1 Table Raw data. This table is relevant data underlying the findings described in manuscript.
(DOCX)
