# Correction: A Retrospective Analysis of the Impact of Myomectomy on Survival in Uterine Sarcoma

**DOI:** 10.1371/journal.pone.0153996

**Published:** 2016-04-14

**Authors:** Zhenzhen Gao, Li’an Li, Yuanguang Meng

An additional affiliation for the first author is not indicated. Zhenzhen Gao is also affiliated with: Department of Obstetrics and Gynecology, Chinese PLA General Hospital, Beijing, China.

## References

[1] Gao Z, Li L, Meng Y (2016) A Retrospective Analysis of the Impact of Myomectomy on Survival in Uterine Sarcoma. PLoS ONE 11(2): e0148050 doi:10.1371/journal.pone.0148050 26828206 10.1371/journal.pone.0148050 PMC4735478

