# Supplementary material for: A Retrospective Analysis of the Impact of Myomectomy on Survival in Uterine Sarcoma
Source: PLoS One. 2016 Feb 1;11(2):e0148050. doi: 10.1371/journal.pone.0148050 (PMC4735478; doi:10.1371/journal.pone.0148050)
Supplement: S1 Table — This table is relevant data underlying the findings described in manuscript. (DOCX) [file pone.0148050.s001.docx]

**Supporting Information**

**S1 Table. Raw data**

| **FIGO stage** | **Grade level** | **Adjuvant therapy** | **Surgical approach** | **Recurrence** | **Outcome** |
| --- | --- | --- | --- | --- | --- |
| I | well differentiated | No | Laparoscopic | no recurrence | subsistence |
| I | well differentiated | No | Laparoscopic | no recurrence | subsistence |
| I | well differentiated | epirubicin+cyclophosphamide | Transvaginal | no recurrence | subsistence |
| III | poorly differentiated | Radiotherapy | Laparoscopic | recurrence | death |
| III | poorly differentiated | No | Transabdominal | recurrence | death |
| I | well differentiated | paclitaxel+carboplatin + radiotherapy | Transvaginal | no recurrence | subsistence |
| I | well differentiated | medroxyprogesterone acetate | Transvaginal | recurrence | subsistence |
| I | well differentiated | medroxyprogesterone acetate | Laparoscopic | no recurrence | subsistence |
| I | poorly differentiated | No | Transvaginal | recurrence | death |
| I | well differentiated | medroxyprogesterone acetate | Transvaginal | recurrence | death |
| I | well differentiated | No | Laparoscopic | no recurrence | subsistence |
| I | well differentiated | No | Transabdominal | no recurrence | subsistence |
| III | well differentiated | No | Transabdominal | no recurrence | subsistence |
| I | well differentiated | No | Laparoscopic | recurrence | death |
| I | well differentiated | No | Transabdominal | recurrence | death |
| I | well differentiated | No | Transabdominal | no recurrence | subsistence |
| II | poorly differentiated | Radiotherapy | Transabdominal | recurrence | death |
| I | poorly differentiated | Radiotherapy | Transvaginal | recurrence | death |
| I | moderately differentiated | Radiotherapy | Transabdominal | no recurrence | subsistence |
| II | well differentiated | No | Transabdominal | recurrence | death |
| II | poorly differentiated | No | Transabdominal | recurrence | death |
| I | moderately differentiated | epirubicin+cyclophosphamide | Transabdominal | recurrence | death |
| I | well differentiated | cyclophosphamide+doxorubicin | Transabdominal | recurrence | death |
| III | well differentiated | paclitaxel+carboplatin +medroxyprogesterone acetate | Transabdominal | no recurrence | subsistence |
| I | poorly differentiated | Radiotherapy | Transabdominal | recurrence | death |
| II | well differentiated | medroxyprogesterone acetate + radiotherapy | Transabdominal | no recurrence | subsistence |
| II | poorly differentiated | Radiotherapy | Transabdominal | recurrence | death |
| I | well differentiated | No | Transvaginal | no recurrence | subsistence |
| II | moderately differentiated | epirubicin+cyclophosphamide | Transvaginal | no recurrence | subsistence |
| III | well differentiated | epirubicin+cyclophosphamide | Transabdominal | no recurrence | subsistence |
| I | well differentiated | No | Transvaginal | no recurrence | subsistence |
| III | moderately differentiated | gemcitabine+cisplatin | Transabdominal | no recurrence | subsistence |
| I | well differentiated | No | Transvaginal | no recurrence | subsistence |
| I | poorly differentiated | Radiotherapy | Transabdominal | no recurrence | subsistence |
| II | poorly differentiated | Radiotherapy | Transabdominal | recurrence | death |
| I | moderately differentiated | carboplatin+epirubicin | Transvaginal | no recurrence | subsistence |
| I | poorly differentiated | Radiotherapy | Transabdominal | no recurrence | subsistence |
| II | poorly differentiated | paclitaxel+cisplatin | Transabdominal | no recurrence | subsistence |
| I | poorly differentiated | No | Transabdominal | recurrence | death |
| I | well differentiated | Radiotherapy | Transvaginal | no recurrence | subsistence |
| I | moderately differentiated | Radiotherapy | Transabdominal | recurrence | death |
| I | well differentiated | medroxyprogesterone acetate + radiotherapy | Transvaginal | recurrence | subsistence |
| I | well differentiated | No | Transabdominal | no recurrence | subsistence |
| I | well differentiated | medroxyprogesterone acetate | Transabdominal | recurrence | death |
| II | well differentiated | No | Transabdominal | recurrence | death |
| I | well differentiated | No | Transvaginal | no recurrence | subsistence |
| I | well differentiated | Radiotherapy | Transvaginal | no recurrence | subsistence |
| I | poorly differentiated | Radiotherapy | Transabdominal | recurrence | death |
| II | moderately differentiated | medroxyprogesterone acetate + radiotherapy | Transabdominal | recurrence | subsistence |
| I | moderately differentiated | No | Transvaginal | recurrence | death |
| I | well differentiated | Radiotherapy | Transvaginal | recurrence | death |
| I | well differentiated | medroxyprogesterone acetate + radiotherapy | Transvaginal | no recurrence | subsistence |
| I | well differentiated | medroxyprogesterone acetate + radiotherapy | Transvaginal | no recurrence | subsistence |
| I | well differentiated | No | Laparoscopic | no recurrence | subsistence |
| I | well differentiated | No | Laparoscopic | no recurrence | subsistence |
| I | moderately differentiated | Radiotherapy | Laparoscopic | no recurrence | subsistence |
| I | well differentiated | No | Transvaginal | no recurrence | subsistence |
| I | well differentiated | No | Laparoscopic | no recurrence | subsistence |
| III | poorly differentiated | No | Laparoscopic | recurrence | subsistence |
